# Dynamic Change of Lumbar Structure and Associated Factors: A Retrospective Study

**DOI:** 10.1111/os.12557

**Published:** 2019-11-03

**Authors:** Jian‐lu Wei, Yan‐bin Zhu, Da‐wang Zhao, Wei Chen, Juan Wang, Hong Wang, Jia‐li Lv, Tao Zhang, Lei Cheng, Ying‐ze Zhang

**Affiliations:** ^1^ Department of Orthopaedic Surgery, Qilu Hospital Shandong University Jinan China; ^2^ Key Laboratory of Biomechanics of Hebei Province Orthopaedic Research Institution of Hebei Province Shijiazhuang China; ^3^ Department of Orthopaedic Surgery the Third Hospital of Hebei Medical University Shijiazhuang China; ^4^ Department of Radiology, Qilu Hospital Shandong University Jinan China; ^5^ Department of Epidemiology and Biostatistics School of Public Health Jinan China

**Keywords:** Aging, Dynamic change, Gender, Kyphosis, Lumbar anatomy parameters

## Abstract

**Objective:**

To determine whether lumbar anatomy parameters are in dynamic change and related factors.

**Methods:**

This is a retrospective study. Participants who did lumbar computed tomography (CT) scanning in Shandong University Qilu Hospital from October 2017 to March 2019 were selected. The 476 participants were randomly selected as male or female, with the age ranging from 17 to 87 years (mean, 55.19; standard deviation, 14.28 years). All the measurements were taken based on the CT scanning image and the measurement of lumbar morphology was conducted using picture archiving and communication systems (PACS). The angle between the horizontal alignment and pedicle center on median sagittal view, the angle between upper endplate and lower endplate on median sagittal view as well as transverse section angle (TSA) using Magerl point in the axial view was determined by reconstructive CT analysis.

**Results:**

In the overall participants, the angle between the horizontal alignment and pedicle center on median sagittal view of lumbar one to three was significantly decreased with aging, from 3.90° ± 2.81° to −4.18° ± 6.86° (*P* = 0.002), 5.60° ± 2.89° to −4.14° ± 5.90° (*P* = 0.030), and 4.75° ± 2.95° to −2.87° ± 4.68° (*P* < 0.001), respectively. Additionally, the angle between the horizontal alignment and pedicle center on median sagittal view in male participants of lumbar two was dramatically decreased, from 4.83° ± 2.79° to −4.45° ± 5.97° (*P* = 0.30). And that of lumbar three in female participants was significantly decreased, from 4.56° ± 2.52° to −2.88° ± 5.03° (*P* = 0.029). Furthermore, of the overall participants, the angle between upper endplate and lower endplate on median sagittal view of lumbar one to four was associated with aging (*P* < 0.001, *P* < 0.001, *P* = 0.015, *P* < 0.001, respectively). The angle of lumbar one, two and four in male participants and lumbar one to four in female participants were all significantly related to aging (all *P* < 0.05). Moreover, in the participants overall, the TSA of lumbar one to three was significantly associated with aging (*P* = 0.015, *P* = 0.006 and *P* = 0.007, respectively). In addition, this angle in lumbar one to lumbar four in male participants were all negatively associated with aging (*P* = 0.017, *P* = 0.001, *P* = 0.005 and *P* = 0.036, respectively).

**Conclusion:**

Lumbar anatomy parameters are in dynamic change in an age and gender dependent manner. During spine surgery in elderly patients, more attention should be paid to these anatomic changes.

## Introduction

The bones in the living body are dynamically changing^1^. Since the seventh week of a human embryo, bones start to form and develop until puberty when they reach maturity^2^. During this period, the shape, mass, bone mineral density, and strength of bone is in dynamic change. It is widely accepted that the bone is changing during this period. For instance, femoral anteversion angle is about 40° in embryonic phase and infancy stage^3^. While this angle is decreased from infancy to adulthood, it finally stays around 15° in adult stage^4^. The neck shaft angle of the femur is about 150° in infancy stage, while this angle turns to be around 125° in adult stage^5^, ^6^, ^7^, ^8^. Besides these, the shape of bones do not remain the same after maturity, and the bone strength, bone mineral density, and bone shape are still changing with aging. Previous studies found the shape of bone – such as diameter, length, and curve – changed accordingly under stress. For instance, a reliable method was established to measure femoral neck torsion angle using femoral neck oblique axial computed tomography (CT) reconstruction^9^, ^10^. By using this method, researchers found that the neck shaft angle of the femur and femoral neck torsion angle of the Asian population were significantly decreased, whereas acetabular anteversion angle was remarkably increased with aging^11^. Additionally, a previous study found that, with aging, medial tibial plateau was decreased and genu varum was accelerated, accompanied by increased proximal fibular curve angle^12^. In the occurrence and progression of degenerative osteoarthritis, the tibial plateau settlement value was increased with the upgrading of Kellgren and Lawrence grades, which is a common method of classifying the severity of knee osteoarthritis^7^. Furthermore, the tibial plateau settlement significantly correlated with the changes of hip‐knee‐ankle angle, minimum medial joint space width, and condylar plateau angle, which are all associated with aging.

Based on previous findings, our group established the “non‐union settlement theory,” which was firstly applied in knee osteoarthritis^12^. We found the ratio of settlement of medial tibial plateau was decreased faster than that of the fibular part. This dynamic change leads to the change of mechanical axis of the lower limb, resulting in more articular cartilage destruction in the medial tibial plateau compartment. Finally, this change causes knee pain and degenerative knee osteoarthritis. Accordingly, our group established an innovative surgery to restore the mechanical axis of the lower limb, which achieves successful outcomes. In addition, this finding promoted us to determine whether the established theory is also applicable in other degenerative diseases.

Denis established “spinal three column theory” in 1983^13^. In this theory, spine stability relies on the structure and balance of three columns (anterior column, medial column, and posterior column). Spine stability is fundamental to realize its function^14^. However, whether its structure remains the same throughout a person's whole life is unclear.

Indeed, in clinic, we found that with aging the incidence of degenerative kyphosis increases^15^. This clinical finding promoted us to hypothesize that lumbar anatomy parameters do not stay the same during the life period. These parameters, including anterior, medial and posterior column, are in dynamic change throughout life. Recent study agreed that degeneration and trauma could induce kyphosis^16^, ^17^; however, whether the exact change in the anatomy parameters initiates kyphosis needs to be further discussed.

Taking osteoarthritis and kyphosis into account, we found some similarity of these two diseases. Firstly, both diseases are accompanied by loading force change. In kyphosis, the sagittal balance is disturbed, leading to changed loading force line. Secondly, they are both degenerative diseases, which have similar pathological processes. It is reported that osteoarthritis and kyphosis are related to osteoporosis; in addition, different levels of osteoporosis lead to non‐union settlement phenomena, causing unbalanced force. Last but not the least, these two diseases may be cured by restoring the balance of the force line. The novelty of this present study is to determine the dynamic change of lumbar parameters from adulthood to older age, which is not focused previously. Additionally, this finding may provide another treatment method for spine degenerative diseases.

Considering the fact that the nature of some bones are still changing, and given the importance of lumbar structure in degenerative kyphosis, herein we determined: (i) whether lumbar anatomy parameters are changing with aging; and (ii) whether the change in lumbar anatomy parameters is associated with gender.

## Materials and Methods

### *Inclusion and Exclusion Criteria*

Each patient provided informed consent for participation in the study. This retrospective study was conducted in accordance with the Declaration of Helsinki (Ethical Principles for Medical Research Involving Human Subjects) and was approved by the Regional Ethics Board of Hebei Medical University (T2016‐001‐1).

This study was designed as a retrospective study and aimed to explore the influences of demographic and physical properties on lumbar morphology. A total of 476 patients (210 male participants and 264 female participants) underwent CT scanning from October 2017 to March 2019. The age of participants ranged from 17 to 87 years, with a mean of 55.19 ± 14.28 years. Three observers examined the lumbar anatomy parameters and recorded them independently. The measurement of lumbar morphology was conducted using picture archiving and communication systems (PACS). Two investigators collected and analyzed data independently.

Inclusion criteria for the study were as follows: (i) ethnic Han patients; (ii) eligible imaging data for measurement; (iii) clear record of age and gender; and (iv) a retrospective study.

Exclusion criteria for the study were as follows: (i) participants with endocrine system diseases (diabetes mellitus, hyperthyroidism, hypothyroidism, Hashimotoʼs thyroiditis, hyperparathyroidism and hypoparathyroidism); (ii) spinal trauma diseases (vertebral compression fractures and vertebral burst fractures); (iii) congenital and developmental anomalies (idiopathic scoliosis and congenital spine structure abnormality); (iv) tumor (abdominal neoplasms and spinal tumor); (v) infections (spinal brucellosis infection, spinal tuberculosis, intervertebral infection, and vertebral infection); (vi) immune system diseases (inflammatory rheumatic arthritis, ankylosing spondylitis, and systemic lupus erythematosus); and /or (vii) clear history of hormone use.

### *Measurement Methods and Parameters*

Qualified participants underwent CT scanning in Qilu Hospital of Shandong University. Reconstructed sagittal and axial view of the CT scan was established in workstation. Three observers did the measurement without detailed information of participants. These observers recorded data independently and directly transferred these data to persons who performed statistical analysis. Statistical analysts collected detailed information and performed data analysis.

### *Clinical Assessment*

#### *Angle Between Upper and Lower Endplate (AULE)*

**Figure 1:**
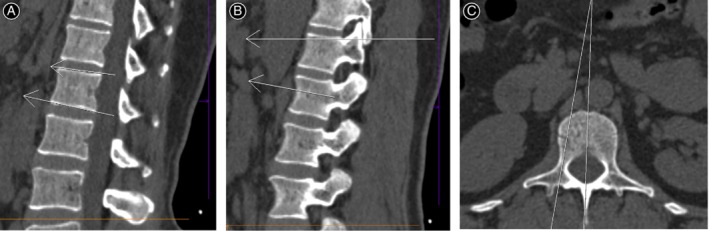
Demonstration of determination of AULE, SSA, and TSA in lumbar. (A) Demonstration of determination of AULE in lumbar. AULE is the angle between the two white arrows. (B) Demonstration of determination of SSA in lumbar. SSA is the angle between the two white arrows. (C) Demonstration of determination of TSA in lumbar. TSA is the angle between the two white arrows.

Definition: in one vertebra body, the angle between the lowest upper endplate and the highest lower endplate. Measurement method: on median sagittal view, we determined the angle between upper endplate and lower endplate of the same vertebra. Clinical significance: this parameter is an indicator of spine sagittal balance. The bigger this angle, the more sever the kyphosis. The present study determined the AULE from lumbar one to lumbar four (Fig. 1A).

#### *Sagittal Section Angle (SSA)*

SSA was defined as the angle between the horizontal alignment and pedicle center, when the pedicle screw was inserted. Measurement method: SSA was examined as previously reported. Briefly, on median sagittal reconstructive view, the angle between the horizontal alignment and pedicle center was recorded as SSA. The angle was recorded as “+” when the pedicle center line is above the horizontal alignment. In turn, we record the angle as “‐” when the pedicle center line is below the horizontal alignment. Clinical significance: this is an important parameter for surgeons to install pedicle screw. In the present study, we examined SSA from lumbar one to lumbar four (Fig. 1B).

#### *Transverse Section Angle (TSA)*

Definition: we positioned the Magerl point, which was defined as the cross point of the tangent to the outer edge of the superior articular process and the medial transverse process. Line “a” was defined as the line that crosses the pedicle center and the Magerl point; and line “b” was defined as the median sagittal view. TSA is the angle between line a and line b. Measurement method: TSA was measured as previously reported^18^, ^19^. Briefly, the cross section of the CT scan image was taken, and TSA was examined as its definition. Clinical significance: this is an important parameter for surgeons to install pedicle screw. The angle must be determined before surgery in case of screw inside of spinal canal or outside of the pedicle. The present study examined TSA from lumbar one to lumbar four (Fig. 1C).

### *Statistical Analysis*

We computed the baseline characteristics (mean, standard deviation) for each gender^20^. Differences in means between participants of different gender were assessed using Studentʼs *t* test or Wilcoxon rank sum test as appropriate^21^. Differences in means between different age groups were assessed using Kruskal‐Wallis *H* test^22^. To explore the correlation between age and pedicle angle, the Pearson product–moment correlation coefficient (*r*) was calculated and scatter plots were shown. Univariable linear regression was used to calculate regression coefficients (β) and *R* ^2^. A *P* value less than 0.05 was judged to be statistically significant. In this study, gender was analyzed as a qualitative variable and other parameters were analyzed as quantitative variables. All analyses were done using R (version 3.5.1, The University of Auckland, New Zealand). Pearson correlation coefficient analysis was used to analyze potential association. Correlation results were interpreted as none or very week (−0.1 to 0.1), weak (−0.3 to −0.1 or 0.1 to 0.3), moderate (−0.5 to −0.3 or 0.3 to 0.5), and strong (−1.0 to −0.5 or 1.0 to 0.5). Multiple linear regression was conducted to assess the independent association. *P* < 0.05 (two‐tailed) was considered statistically significant. A highly significant difference was defined as *P* < 0.01 (two‐tailed).

## Results

### *Lumbar Anatomy Parameters are Changing with Aging*

To investigate whether lumbar anatomy parameters are changing throughout life, we analyzed participants from 17 years to 87 years of age. As demonstrated in Table 1, we divided the population into five groups and all information was included. As shown in Table 2, there was significant difference of AULE in lumbar one in different age stages, from 8.07° ± 3.06° to 4.29° ± 2.40° (*P* = 0.032). Additionally, SSA of lumbar one (from 3.90° ± 2.81° to −4.18° ± 2.86°, *P* = 0.002), lumbar two (from 5.60° ± 2.89° to −4.14° ± 5.90°, *P* = 0.030) and lumbar three (from 4.75° ± 2.95° to −2.87° ± 4.68°, *P* < 0.001) in different age stages also showed a remarkable difference.

**Table 1:** Data distribution

Index	Mean	W	*P* value	*β*	95%*CI*
Age (yeas)	60 (44.25, 66.00)	0.9587	<0.001		
L_1_ AULE	0.87 (0.83, 0.92)	0.9618	<0.001	−0.0666	−0.0874, −0.0458
L_1_ SSA	−1.89 (6.49)	0.9946	0.095	−0.2123	−0.2473, −0.1774
L_1_ TSA	−1.95 (−6.30, 2.93)	0.9706	<0.001	−0.0107	−0.0194, −0.0021
L_2_ AULE	3.7 (1.8, 5.8)	0.9516	<0.001	−0.0427	−0.0582, −0.0271
L_2_ SSA	−3.35 (−7.30, 3.90)	0.9836	<0.001	−0.2684	−0.3051, −0.2316
L_2_ TSA	7.45 (6.40, 8.70)	0.9687	<0.001	−0.0121	−0.0220, −0.0023
L_3_ AULE	2.2 (1.1, 4.4)	0.9069	<0.001	−0.0179	−0.0323, −0.0034
L_3_ SSA	0.0 (−4.6, 3.1)	0.9924	0.017	−0.1788	−0.2091, −0.1484
L_3_ TSA	8.7 (7.6, 10.0)	0.9756	<0.001	−0.0153	−0.0264, −0.0043
L_4_ AULE	2.50 (0.30,5.00)	0.962	<0.001	0.0535	0.0325, 0.0744
L_4_ SSA	3.00 (0.08, 5.60)	0.9732	<0.001	−0.0126	−0.0376, 0.0125
L_4_ TSA	10.35 (8.93, 12.00)	0.2692	<0.001	−0.0101	−0.0237, 0.0036

AULE, angle between upper and lower endplate; SSA, sagittal section angle; TSA, transverse section angle.

Shapiro–Wilk test was used.

**Table 2:** The basic information of AULE, SSA and TSA of all participants (mean (SD), °)

Index	17–31 years	31–45 years	45–59 years	59–73 years	73–87 years	*P* value
L_1_ AULE	8.07 (3.06)	7.39 (3.27)	4.77 (3.11)	5.33 (3.44)	4.29 (2.40)	0.032
L_1_ SSA	3.90 (2.81)	3.16 (3.42)	−2.22 (5.87)	−4.49 (5.97)	−4.18 (6.86)	0.002
L_1_ TSA	6.91 (1.11)	7.19 (1.29)	6.92 (1.41)	6.78 (1.32)	6.73 (1.40)	0.055
L_2_ AULE	5.21 (3.03)	4.98 (2.62)	3.48 (2.47)	3.65 (2.37)	2.80 (2.00)	0.219
L_2_ SSA	5.60 (2.89)	4.73 (4.51)	−3.24 (5.84)	−5.29 (5.94)	−4.14 (5.90)	0.030
L_2_ TSA	7.70 (1.44)	7.93 (1.54)	7.53 (1.57)	7.45 (1.49)	7.45 (1.66)	0.310
L_3_ AULE	2.34 (2.31)	3.43 (2.61)	2.59 (2.28)	2.64 (2.05)	2.38 (2.14)	0.447
L_3_ SSA	4.75 (2.95)	4.10 (3.76)	−1.53 (4.34)	−2.67 (5.33)	−2.87 (4.68)	<0.001
L_3_ TSA	8.84 (1.53)	9.17 (1.72)	8.79 (1.76)	8.74 (1.78)	8.42 (2.04)	0.065
L_4_ AULE	0.46 (2.40)	1.72 (3.33)	2.62 (3.39)	3.38 (2.90)	2.93 (3.95)	0.337
L_4_ SSA	3.75 (2.81)	3.02 (2.45)	3.65 (3.51)	2.88 (4.57)	2.84 (4.65)	0.270
L_4_ TSA	10.54 (2.09)	10.84 (1.96)	10.28 (2.25)	10.36 (2.10)	10.41 (2.17)	0.131

AULE, angle between upper and lower endplate; SSA, sagittal section angle; TSA, transverse section angle.

Shapiro–Wilk test was used.

To further determine whether this difference is gender‐affected, we analyzed each parameter separately. As illustrated in Table 3, male SSA in lumbar two exhibited significant difference in different age stages (from 4.83° ± 2.79° to −4.45° ± 5.97°, *P* = 0.030). As shown in Table 4, female SSA in lumbar three demonstrated significant difference in different age stages (from 4.56° ± 2.52° to −2.88° ± 5.03°, *P* = 0.029).

**Table 3:** The basic information of AULE, SSA, and TSA of male participants

Index	17–31 years	31–45 years	45–59 years	59–73 years	73–87 years	*P* value
L_1_ AULE	9.52 (2.88)	8.09 (3.24)	5.51 (2.41)	6.17 (3.34)	4.48 (2.20)	0.096
L_1_SSA	3.41 (2.27)	2.84 (2.87)	−1.40 (4.07)	−4.07 (5.76)	−4.21 (5.93)	0.119
L_1_TSA	7.06 (1.21)	7.15 (1.27)	7.10 (1.35)	6.73 (1.23)	6.55 (1.67)	0.131
L_2_ AULE	5.65 (3.36)	5.02 (2.78)	4.25 (2.77)	3.78 (2.63)	2.66 (2.18)	0.355
L_2_SSA	4.83 (2.79)	4.50 (3.87)	−2.13 (4.21)	−5.20 (6.62)	−4.45 (5.97)	0.030
L_2_TSA	8.06 (1.42)	7.96 (1.48)	7.65 (1.44)	7.39 (1.41)	6.95 (1.52)	0.321
L_3_ AULE	2.42 (1.70)	3.15 (2.88)	2.78 (2.22)	2.59 (2.21)	2.31 (2.19)	0.347
L_3_SSA	4.89 (3.32)	4.42 (3.26)	−1.02 (3.18)	−2.78 (5.52)	−2.85 (4.40)	0.088
L_3_TSA	9.16 (1.43)	9.08 (1.53)	8.82 (1.67)	8.53 (1.58)	7.91 (1.56)	0.326
L_4_ AULE	0.47 (2.51)	1.27 (3.44)	2.09 (3.66)	2.45 (2.97)	1.85 (4.28)	0.798
L_4_SSA	3.75 (3.11)	3.49 (2.39)	3.48 (2.91)	2.26 (4.31)	2.99 (4.56)	0.138
L_4_TSA	10.67 (1.65)	10.66 (1.92)	10.48 (2.17)	10.13 (1.93)	9.72 (1.99)	0.450

AULE, angle between upper and lower endplate; SSA, sagittal section angle; TSA, transverse section angle.

Shapiro–Wilk test was used.

**Table 4:** The basic information of AULE, SSA, and TSA of female participants

Index	17–31 years	31–45 years	45–59 years	59–73 years	73–87 years	*P* value
L_1_ AULE	6.03 (2.02)	6.57 (3.15)	4.35 (3.39)	4.70 (3.40)	4.13 (2.60)	0.081
L_1_SSA	4.58 (3.44)	3.54 (3.96)	−2.68 (6.65)	−4.81 (6.13)	−4.14 (7.73)	0.386
L_1_TSA	6.74 (1.02)	7.24 (1.34)	6.82 (1.38)	6.87 (1.16)	6.82 (1.44)	0.364
L_2_ AULE	4.64 (2.59)	4.95 (2.43)	3.00 (2.16)	3.54 (2.14)	2.95 (1.86)	0.426
L_2_ SSA	6.69 (2.79)	5.01 (5.20)	−3.85 (6.53)	−5.36 (5.38)	−3.87 (6.01)	0.071
L_2_ TSA	7.28 (1.42)	7.91 (1.64)	7.47 (1.65)	7.50 (1.54)	7.83 (1.69)	0.363
L_3_ AULE	2.23 (3.10)	3.77 (2.20)	2.48 (2.32)	2.68 (1.92)	2.45 (2.17)	0.576
L_3_ SSA	4.56 (2.52)	3.72 (4.29)	−1.82 (4.86)	−2.59 (5.19)	−2.88 (5.03)	0.029
L_3_ TSA	8.45 (1.63)	9.29 (1.92)	8.77 (1.83)	8.90 (1.90)	8.79 (2.30)	0.078
L_4_ AULE	0.44 (2.36)	2.33 (3.13)	2.94 (3.22)	4.09 (2.65)	3.94 (3.44)	0.467
L_4_ SSA	3.75 (2.50)	2.48 (2.42)	3.75 (3.83)	3.37 (4.72)	2.68 (4.89)	0.557
L_4_ TSA	10.40 (2.58)	11.06 (2.01)	10.17 (2.29)	10.53 (2.21)	10.92 (2.21)	0.179

AULE, angle between upper and lower endplate; SSA, sagittal section angle; TSA, transverse section angle.

Shapiro–Wilk test was used.

### *Lumbar Anatomy Parameters are Correlated to Age*

We further determined the correlation between aging and lumbar parameters despite other factors. As indicated in Table 5, AULE of lumbar one to lumbar four was correlated to aging, which showed a significant statistical difference for each level (*P* < 0.001, *P* < 0.001, *P* = 0.015, *P* < 0.001, respectively). Respectively, AULE of lumbar one to lumbar three was decreased as age increased. However, AULE of lumbar four was increased as age increased. SSA of lumbar one to lumbar three showed significant difference in different age stages (*P* < 0.001, *P* < 0.001, *P* < 0.001, respectively), indicating SSA of lumbar one to lumbar three was negatively associated with age. Additionally, TSA of lumbar one to lumbar three illustrated negative relation to age (*P* = 0.015, *P* = 0.006 and *P* = 0.007).

**Table 5:** Correlation between age and AULE, SSA, or TSA of all participants

Locations	AULE			SSA			TSA		
	*r*	*b*	*P* value	*r*	*b*	*P* value	*r*	*b*	*P* value
L_1_	−0.2798	−0.0666	<0.001	−0.4847	−0.2123	<0.001	−0.1128	−0.0107	0.015
L_2_	−0.2476	−0.0427	<0.001	−0.5532	−0.2684	<0.001	−0.1288	−0.0138	0.006
L_3_	−0.1147	−0.0179	0.015	−0.4738	−0.1788	<0.001	−0.1263	−0.0153	0.007
L_4_	0.2371	0.0535	<0.001	−0.0468	−0.0126	0.324	−0.0674	−0.0101	0.148

AULE, angle between upper and lower endplate; SSA, sagittal section angle; TSA, transverse section angle.

Shapiro–Wilk test was used.

### *Lumbar Anatomy Parameters are Age‐Dependent and Gender‐Dependent*

Given the importance that men and women have different hormone changes which may affect bone change, we further investigated whether the dynamic change in lumbar is gender‐dependent. To address this issue, these data of two genders was analyzed respectively.

**Figure 2:**
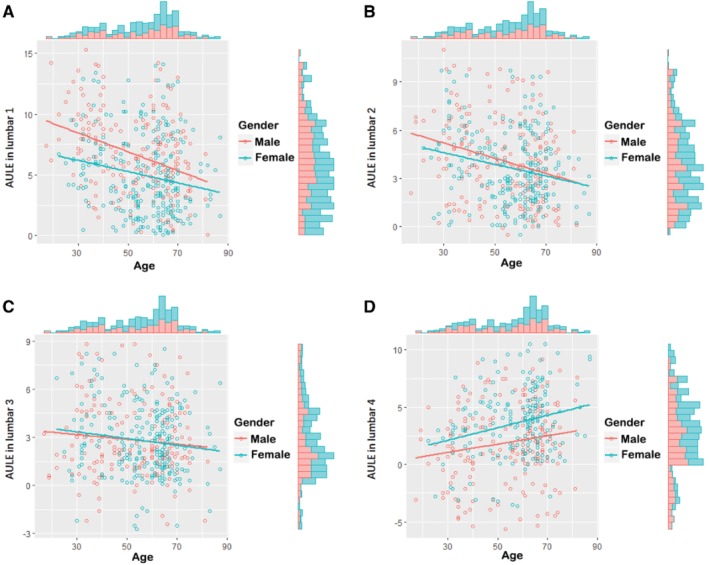
AULE in lumbar one to lumbar four. (A) AULE in lumbar one of all participants. (B) AULE in lumbar two of all participants. (C) AULE in lumbar three of all participants. (D) AULE in lumbar four of all participants. Red dot and line present male participants, blue dot and line present female participants. Columns present population.

As indicated in Table 6 and Fig. 2, male AULE in lumbar one (*P* < 0.001), lumbar two (*P* < 0.001) and lumbar four (*P* = 0.020) was correlated to age. In detail, male AULE in lumbar one (*r* = −0.3510) and lumbar two (*r* = −0.2728) was decreased with aging, while male AULE in lumbar four (*r* = 0.1663) was increased with aging.

**Table 6:** Correlation between age and AULE, SSA, or TSA of male participants

Locations	AULE			SSA			TSA		
	*r*	*b*	*P* value	*r*	*b*	*P* value	*r*	*b*	*P* value
L_1_	−0.3510	−0.0775	<0.001	−0.5296	−0.1978	<0.001	−0.1656	−0.0149	0.017
L_2_	−0.2728	−0.0511	<0.001	−0.5840	−0.2657	<0.001	−0.2207	−0.0220	0.001
L_3_	−0.0828	−0.0130	0.242	−0.5269	−0.1919	<0.001	−0.1971	−0.0213	0.005
L_4_	0.1663	0.0361	0.020	−0.1123	−0.0270	0.110	−0.1472	−0.0199	0.036

AULE: Angle between upper and lower endplate; SSA: Sagittal section angle; TSA: transverse section angle.

Shapiro–Wilk test was used.

**Figure 3:**
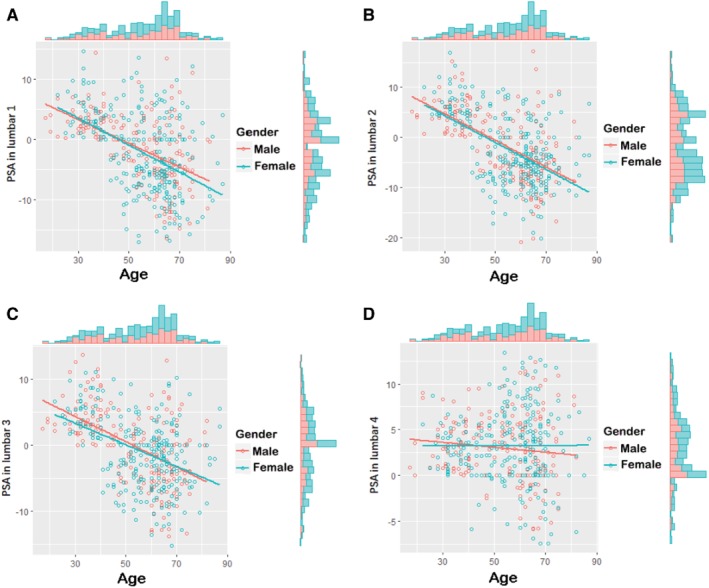
SSA in lumbar one to lumbar four. (A) SSA in lumbar one of all participants. (B) SSA in lumbar two of all participants. (C) SSA in lumbar three of all participants. (D) SSA in lumbar four of all participants. Red dot and line present male participants, blue dot and line present female participants. Columns present population.

Additionally, as indicated in Table 6 and Fig. 3, male SSA in lumbar one to lumbar three was associated with aging (*P* < 0.001, *P* < 0.001, *P* < 0.001, respectively). This association was negatively related (*r* = −0.5296, *r* = −0.5840, *r* = −0.5269, respectively).

**Figure 4:**
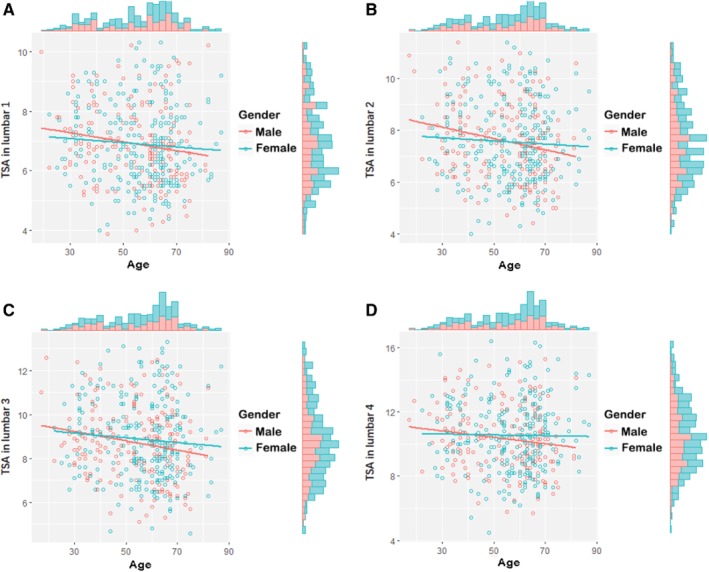
TSA in lumbar one to lumbar four. (A) TSA in lumbar one of all participants. (B) TSA in lumbar two of all participants. (C) TSA in lumbar three of all participants. (D) TSA in lumbar four of all participants. Red dot and line present male participants, blue dot and line present female participants. Columns present population.

Furthermore, as indicated in Table 6 and Fig. 4, male TSA in lumbar one to lumbar four was negatively associated with age for each lumbar level (*P* = 0.017, *P* = 0.001, *P* = 0.005, *P* = 0.036, respectively).

As indicated in Table 7 and Fig. 2, female AULE in lumbar one to lumbar four was correlated to age for each lumbar level (*P* = 0.003, *P* < 0.001, *P* = 0.041, *P* < 0.001, respectively). Respectively, female AULE in lumbar one to lumbar three was decreased with aging (*r* = −0.1811, *r* = −0.2158, *r* = −0.1302, respectively), while female AULE in lumbar four was increased with aging (*r* = 0.2752).

**Table 7:** Correlation between age and AULE, SSA, or TSA of female participants

Locations	AULE			SSA			TSA		
	*r*	*b*	*P* value	*r*	b	P value	*r*	*b*	*P* value
L_1_	−0.1811	−0.0446	0.003	−0.4544	−0.2281	<0.001	−0.0698	−0.0070	0.264
L_2_	−0.2158	−0.0364	<0.001	−0.5222	−0.2684	<0.001	−0.0279	−0.0033	0.655
L_3_	−0.1302	−0.0210	0.041	−0.4215	−0.1694	<0.001	−0.0247	−0.0034	0.693
L_4_	0.2752	0.0619	<0.001	0.0075	0.0022	0.908	−0.0159	−0.0026	0.800

AULE, angle between upper and lower endplate; SSA, sagittal section angle; TSA, transverse section angle.

Shapiro–Wilk test was used.

Moreover, as shown in Table 7 and Fig. 3, female SSA in lumbar one to three was negatively associated with age (*P* < 0.001, respectively).

However, as shown in Table 7 and Fig. 4, female TSA from lumbar one to four did not show any correlation with age (*P* = 0.264, *P* = 0.655, *P* = 0.693, *P* = 0.800, respectively).

Collectively, the change of lumbar anatomy parameters was associated with age and this association was gender‐dependent.

## Discussion

Previous study found some bones were not unchanged with aging^12^, ^23^. For example, femoral anteversion angle and neck shaft angle of the femur were changing with aging. And tibial curve was also related to aging, as well as osteoarthritis progression. These previous works were first claimed by our group and researchers realized bone was still changing from the so‐called mature stage to the aging stage. Importantly, based on these findings, a new surgery method was invented for the degenerative osteoarthritis and got the relative satisfied outcome^12^. The phenomenon of bone changing with aging also reminded surgeons to take appropriate actions for the specific individual. Herein, the present study proposed that, with aging, spinal structure was changed and degenerated, leading to an imbalance of the established structure, leading to kyphosis.

In the present study, we found that all of the lumbar anatomy parameters were correlated to age. In addition, this correlation was age‐dependent and gender‐dependent. Importantly, male and female participants did not show the exact same change. In detail, in both genders, AULE in lumbar one and lumbar two was decreased with aging, while AULE in lumbar four was increased with aging. In addition, in both genders, SSA in lumbar one to lumbar three was negatively associated with age. However, male AULE in lumbar three showed no significant difference while female AULE in lumbar three showed significant difference. Moreover, male TSA in lumbar one to lumbar four was negatively associated with age, while female TSA in lumbar one to lumbar four demonstrated no significant difference. Collectively, these data indicated that, with increased age, body structure also changed in accordance to required force balance, and that structural change may be affected by gender.

Our group established the non‐uniform settlement theory by researching anatomy, radiology, biomechanics, and clinic^12^, ^24^. We proposed osteoporosis was the initial factor for causing force change. Osteoporosis is a systemic disease which is characterized by decreased bone mass, destructed bone microstructure, and increased osteopsathyrosis^25^. Osteoporosis is related to biomechanics loading, hormone and local biochemistry cytokines^26^, ^27^, ^28^. Osteoporosis is a common disease which is predicted to affect 400 mn people, accounting for 27% of the whole population^29^. The world is stepping into an aging society, in which osteoporosis increases mortality by evoking fractures and their complications. However, supplements of calcium tablets, vitamin D, or both cannot effectively reduce osteoporosis‐induced fractures^30^.

In the musculoskeletal system, bone plays an important role in maintaining position by its supporting function^31^. However, with aging, osteoclasts are activated and proliferated, while osteoblasts are inactivated, causing decreased bone mineral density and strength, leading to osteoporosis^32^. In this situation, the established structure cannot hold existed body weight. In order to achieve the supporting ability, lumbar structure is changed accordingly.

Degenerative kyphosis is partially caused by osteoporosis^33^. With aging, osteoporosis occurs, leading to loss of bone mass. The osteoporotic bone cannot support previous loading, causing microfracture of the vertebral trabecula, leading to the change of vertebra shape. Accordingly, the lumbar anatomy parameters change to adapt to its structure. Consequently, these changes lead to degenerative diseases, including kyphosis, spinal lumbar stenosis, and scoliosis. In the present study, we found that, with aging, the AULE in lumbar one was significantly different (Table 2). The decreased angle indicated upgrading lumbar lordosis (LL), showing increased curve in spine, leading to kyphosis. Furthermore, TSA in lumbar one to lumbar three demonstrated significant difference (Table 5) in the present study. The TSA in lumbar one to lumbar three was decreased with aging. In this situation, the spinal canal was narrowed, leading to lumbar spinal stenosis. Importantly, the degenerative scoliosis was commonly found in lumbar two and lumbar three, and we found the lumbar anatomy parameters were statistically difference in lumbar one to three. Our finding is consistent with previous findings to some extent.

Non‐uniform settlement theory was firstly applied in osteoarthritis, and the present study found degenerative kyphosis also had the non‐uniform settlement phenomenon. Firstly, decreased AULE indicated that anterior vertebral body lost more height than posterior vertebral body. Secondly, the compacted vertebral body had the ability to afford body loading. Furthermore, the lumbar anatomy parameters, including anterior, medial, and posterior, all changed with aging. Importantly, this pathological process is chronic, finally leading to degenerative diseases. Notably, restoring body biomechanics may be a potential treatment for these mentioned diseases.

Our group previously established “non‐uniform settlement phenomenon” in osteoarthritis and “dynamic change” in the hip. These two findings are correlated and in consequence. Since bone suffers from different loading and osteoporosis occurs differently, the shape, bone mineral density, and strength of the bone all change to adapt to its nature. In this process, the bone is in dynamic change, and finally we found this non‐uniform settlement phenomenon. In the present study, we found lumbar anatomy parameters were in dynamic change, however, whether this change was induced by osteoporosis needs to be further discussed.

The present study also had some limitations. First of all, this is retrospective study, and more information should be discussed in the follow‐up prospective study in order to exclude the effect of other factors. Secondly, the mechanism and initiation factor of kyphosis were not investigated in the present study.

Collectively, the height of anterior column of lumbar one was decreased with aging which is associated with kyphosis, and the TSA in lumbar one, lumbar two and lumbar three was negatively associated with age. In turn, this association is gender‐dependent. Spinal surgeons should pay attention to these changes when performing the related operation.
